# ViaFuse: Fiji macros to calculate skeletal muscle cell viability and fusion index

**DOI:** 10.1186/s13395-021-00284-3

**Published:** 2021-12-16

**Authors:** Emma Rose Hinkle, Tasneem Omar Essader, Gabrielle Marie Gentile, Jimena Giudice

**Affiliations:** 1 Department of Cell Biology and Physiology, School of Medicine, The University of North Carolina at Chapel Hill, Chapel Hill, NC 27599 USA; 2 Curriculum in Genetics and Molecular Biology (GMB), The University of North Carolina at Chapel Hill, Chapel Hill, NC 27599 USA; 3 McAllister Heart Institute, School of Medicine, The University of North Carolina at Chapel Hill, Chapel Hill, NC 27599 USA

**Keywords:** Skeletal muscle, Myogenesis, Skeletal muscle cell software, C2C12 cell differentiation quantification, Fusion index

## Abstract

**Background:**

Measuring biological features of skeletal muscle cells is difficult because of their unique morphology and multinucleate nature upon differentiation. Here, we developed a new Fiji macro package called ViaFuse (that stands for viability and fusion) to measure skeletal muscle cell viability and differentiation. To test ViaFuse, we utilized immunofluorescence images of differentiated myotubes where the capping actin protein of muscle z-line subunit beta (CAPZB) was depleted in comparison with control cells.

**Results:**

We compared the values achieved using the ViaFuse macros first with manual quantification performed by researchers and second with those obtained utilizing the MATLAB muscle-centric software MyoCount. We observed a high degree of correlation between all methods of quantification.

**Conclusions:**

ViaFuse can detect the borders of myotubes and identify nuclear clumps which have been limitations of previous muscle-centric imaging software. The ViaFuse macros require little computer power or space to run and user inputs to the ViaFuse macros are minimal, thereby automating the analysis process in a quick, easy, and accurate fashion. Additionally, the ViaFuse macros work with Fiji, an existing imaging software widely used by skeletal muscle researchers. Furthermore, ViaFuse is compatible with many computer systems, has a very intuitive interface, and does not require prior complex mathematical knowledge. Therefore, we propose ViaFuse as a robust and meticulous method to quantify skeletal muscle cell viability and differentiation.

**Supplementary Information:**

The online version contains supplementary material available at 10.1186/s13395-021-00284-3.

## Background

Skeletal muscle cells are easy to culture and thus are often utilized to study different molecular and cellular aspects of myogenesis [1]. Characteristics like cell viability and fusion can be analyzed to determine the role of specific proteins, culturing conditions, or stimuli on the myogenic process. For example, a low viability rate after protein depletion may indicate its involvement in cell growth (or apoptosis). Skeletal muscle cells go through differentiation where mononucleated myoblasts fuse with each other to form elongated, multinucleated myotubes [2]. Muscle cell differentiation can be estimated by measuring the fusion index, which is the percentage of nuclei within differentiated myotubes.

It is frequently difficult to manually quantify the characteristics of skeletal muscle cells in a dependable and error-free manner because their morphology can vary widely depending on differentiation stage. It is important that quantifications are accurate and represent what is biologically observed. Some labs utilize various stains to determine myotube density, but these methods are limited to experiments where all myotubes exhibit similar morphology [3, 4]. Numerous groups manually quantify microscopy images to estimate cell viability and fusion since most software cannot accurately segment between differentiated myotubes because they group close together, are long, and multinucleated [3, 5–8]. However, there are limitations to manual quantification. First, it can vary depending on the researcher and it needs to be performed in a blinded manner. Second, it is very time-consuming, especially considering that for proper accuracy and statistical analysis, numerous images need to be analyzed. Thus, there is a real need for a muscle-centric software that can measure various features of skeletal muscle cells in a consistent way that minimizes human error and takes a reasonable amount of time [9, 10].

There is currently one available muscle-centric software tool (MyoCount) [11] that offers a method to standardize and automate the quantification of myoblast fusion. MyoCount is a MATLAB-based software that provides users with a list of adjustable parameters for identifying nuclei and masking myotubes, allowing for optimization of the analysis based on image quality. The ability to perform high-throughput image processing while also reducing human bias are clear advantages of using MyoCount over manual image quantifications. However, issues persist with inaccurate counting of clustered nuclei, distinguishing a myoblast from a close myotube, outlining complete myotubes, and determining myoblast and myotube borders correctly when they are close to each other. The MyoCount software computing power is also limited by image size, and it can be a challenge for users to modify the source code if they do not have very deep knowledge of the MATLAB platform.

Here, we present ViaFuse (viability and fusion), an accessible Fiji (Fiji is just ImageJ) macro package that quickly, intuitively, and accurately quantifies skeletal muscle cell viability (ViaFuse viability, ViaFuse-V) and differentiation of myoblasts into myotubes (ViaFuse fusion, ViaFuse-F). We compared ViaFuse quantifications of CAPZB-depleted myotubes to those performed manually and with MyoCount and observed high degrees of correlation between the methods. Thus, we conclude that ViaFuse is a powerful tool to analyze skeletal muscle cell viability and differentiation that has several advantages: (i) its ease of use for researchers, (ii) its ability to distinguish and account for clumps of nuclei, (iii) its capacity to identify myotube borders correctly, (iv) its capacity to distinguish nearby myoblasts as multiple unfused myoblasts and not as one myotube, and (v) its minimal equipment requirement.

## Implementation

### C2C12 cell culture

Cells were cultured in growth medium composed of Dulbecco’s modified Eagle’s medium (DMEM), 10% fetal bovine serum, 2 mM l-glutamine, and 0.01% penicillin and streptomycin. When cells reached 90% confluency, the media was switched to differentiation media which was composed of DMEM, 2% horse serum, 2 mM l-glutamine, and 0.01% penicillin and streptomycin.

### Delivery of small interfering RNAs (si-RNAs) via electroporation

C2C12 myoblast cells were transfected with two different Capzb si-RNAs from Dharmacon (si-Capzb-#1: #MQ-040494-01-0002 and si-Capzb-#2: #MQ-040494-01-0004) or the si-GENOME negative control also from Dharmacon (#D-001210-01-05), all at a final concentration of 87 pmol/mL. The Neon electroporation system (Thermo Fisher Scientific, MPK10096) was used to deliver the si-RNAs into C2C12 myoblasts according to the manufacturer instructions: 750,000 cells per well were electroporated using a 100 μL Neon pipette with conditions of 1650 V, width of 3 ms, and three pulses. The next day (24 h after electroporation), myoblasts were induced to differentiate into myotubes. After 4 days of differentiation, protein lysates were extracted from the cells or the cells were fixed, immunostained, and visualized by confocal microscopy.

### Protein lysate preparation

Cells were placed on ice, washed with cold PBS, and lysed using ice cold RIPA buffer (50 mM Tris, 120 mM NaCl, 1% Triton X-100, 0.1% sodium dodecyl sulfate (SDS), 0.5% sodium deoxycholate) containing protease (Roche, #11873580001) and phosphatase (Roche, #04906845001) inhibitors. Lysates were incubated on ice for 15 min, then sonicated at 75% amplitude (intensity) for 3 min (30 s on and 30 s off). The lysates were incubated on ice for 15 min and then centrifuged at 14,000 rpm for 10 min at 4 °C. The supernatants were transferred into new tubes and stored at − 80 °C. Protein concentration was measured using the Pierce BCA protein assay kit (Thermo Fisher Scientific, #23225).

### Western blotting assays

Protein lysates (30 μg) were analyzed by western blotting to verify efficient CAPZB depletion. Samples were analyzed by polyacrylamide gel electrophoresis (SDS-PAGE) on a 12% mini-Protean TGX stain-free gel (BioRad, #4568044). The running buffer consisted of 200 mM glycine, 25 mM Tris base, and 3.5 mM SDS. Electrophoresis was performed at 90 V for 30 min and then 150 V for another 30 min. Gels were imaged using the ChemiDoc Imaging System from BioRad. The proteins were then transferred from the gel to an Amersham Hybond Low Fluorescence 0.2 μm PVDF membrane (GE Healthcare Life Sciences, #10600022) at 100 V for 1 h in transfer buffer (100 mM glycine, 25 mM Tris, and 20% methanol). Total proteins in the membranes were visualized using the ChemiDoc Imaging System from BioRad. Membranes were then blocked for 1 h with 5% nonfat dry milk in Tris-buffered saline buffer containing tween 20 (TBST) (400 mM Tris base, 2.74 M NaCl, 0.1% tween 20, pH 7.6). Membranes were briefly washed with TBST and then incubated with the appropriate primary antibodies diluted in TBST overnight at 4 °C: mouse anti-LMNA (Santa Cruz Biotechnologies, sc-7293, dilution 1:500), rabbit anti-CAPZB (Abcam, ab220669, dilution 1:100). The following day, membranes were washed with TBST three times (10 min each) and then incubated for 1 h with the secondary goat anti-mouse antibody Dylight 800 from Thermo Fisher Scientific (SA5–35521, dilution 1:10,000). Membranes were imaged using an Odyssey LiCor Imager.

### Immunofluorescence assays

On the fourth day of differentiation, cells were fixed for 2 min in a 2% paraformaldehyde solution in cell medium (a 4% paraformaldehyde solution in PBS—made from EMS 16% Paraformaldehyde Aqueous Solution EM Grade, Fisher Scientific, #15710—was diluted in half with cell culture medium). The media containing the paraformaldehyde solution was aspirated, and cells were then incubated with 2% paraformaldehyde in PBS for 20 min. Cells were washed with PBS three times (10 min each) and then blocked in an immunofluorescence buffer (BIF) containing PBS, 1% bovine serum albumin, and 0.3% Triton for 1 h at room temperature. Cells were next incubated with the mouse polyclonal IgG anti-myosin heavy chain (MYH) 3 (Santa Cruz Biotechnologies, sc-53091) diluted (1:50) in BIF overnight at 4 °C. The following day, cells were washed with PBS three times (10 min each) and then incubated for 1 h in the darkness and at room temperature with an anti-mouse antibody conjugated with Alexa Fluor 488 (AlexaFluor 488((ab)) 2 fragment of goat anti-mouse IgG (HHL), Thermo Fisher Scientific, A1101) diluted (1:500) in BIF. Cells were then washed with PBS three times (10 min each) and incubated with 2 μM DAPI diluted in PBS for 5 min at room temperature. Cells were washed twice with PBS and imaged in PBS immediately.

### Confocal microscopy

Cells were imaged in the Hooker Imaging Core at The University of North Carolina at Chapel Hill using a Zeiss LSM 880 confocal microscope with a 10x Plan Apochromat objective (0.45 WD) and the Zen Black (Zeiss) software was utilized for image acquisition. Stitched images (3 × 3) were taken of each condition. The following excitation parameters were used: argon multiline laser at 488 nm (for Alexa Fluor 488) and a 405 nm diode at 30 mW (for DAPI). Cells were imaged at room temperature.

### Manual image analysis

The captured images were analyzed using Fiji software. The images were unstacked and DAPI was assigned a red color and MYH was assigned a green color. DAPI and MYH images were multiplied variably to ensure all images looked similar for quantification. To calculate the total number of nuclei, a threshold was first applied to the DAPI image to make it binary, small holes were then filled and a watershed separation was utilized to separate close nuclei. The area of nuclei sized between 50 μm^2^ and 250 μm^2^ and those greater than 251 μm^2^ was procured using the analyze particles tool. To determine the number of nuclei in nuclear clumps, the areas of the objects larger than 251 μm^2^ were divided by the mode of the nuclei areas between 50 μm^2^ and 250 μm^2^. The obtained number of nuclei in nuclear clumps was then added to the number of nuclei with areas between 50 μm^2^ and 250 μm^2^ to generate the total number of nuclei in an image. Finally, the total number of nuclei in the image was divided by the image size (in mm^2^) to procure the total number of nuclei per unit area (nuclei/mm^2^). For calculation of the fusion index, the DAPI and MYH images were merged. Using the grid and count cells plugin, the nuclei that were found outside of MYH-positive cells and those in MYH-positive cells with one or two nuclei were counted. Fusion index was thus calculated using the following equation:

$$\mathrm{Fusion}\ \mathrm{in}\mathrm{dex}=\frac{\mathrm{total}\ \mathrm{nuclei}-\mathrm{nuclei}\ \mathrm{outside}\ \mathrm{MYH}\ \mathrm{positive}\ \mathrm{cells}-\mathrm{nuclei}\ \mathrm{in}\ \mathrm{MYH}\ \mathrm{positive}\ \mathrm{cells}\ \mathrm{with}\ 1\ \mathrm{or}\ 2\ \mathrm{nuclei}}{\mathrm{total}\ \mathrm{nuclei}}\times 100$$

### Operation and guidelines to install ViaFuse macros

To run the ViaFuse macros, the computer must be a Windows XP or later, a Mac OS X 10.8 “Mountain Lion” or later, or a Linux on amd64 and × 86 architectures. The system must also run Java 8 or later. To install Fiji, the following steps should be followed: (1) navigate to https://Fiji.sc/, (2) scroll to the “Downloads” section and download the correct version of Fiji for the computer (the following macros were made with version 1.53c for MacOS and version 1.53 h for Windows), and (3) follow Fiji installation instructions and ensure that the correct version is installed.

To download the ViaFuse macros, two steps should be followed: (1) navigate to https://github.com/tasneemsmacros/ViaFuse and (2) download the desired macros (ViaFuse-V or ViaFuse-F) for your operating system and save them to your computer. Instructions for each macro are available for download at that link as well.

### ViaFuse-V, macros to calculate total nuclei

ViaFuse-V uses the DAPI image to generate a .csv file with (i) a list of the areas of particles with sizes greater than 251 μm^2^, (ii) the number of nuclei sized between 50 μm^2^ and 250 μm^2^, (iii) the mode of nuclei sized between 50 μm^2^ and 250 μm^2^, and (iv) the area of the image (in mm^2^). To calculate the number of nuclei in clumps, the user divides the sizes of particles greater than 251 μm^2^ (generated by ViaFuse-V) by the mode of nuclei sized between 50 μm^2^ and 250 μm^2^ (generated by ViaFuse-V). This number can be added to the number of nuclei sized 50 μm^2^ and 250 μm^2^ (generated by ViaFuse-V) to produce the total nuclei number in an image. The total nuclei number is then divided by the image size measured in mm^2^ (generated by ViaFuse-V) to calculate the total number of nuclei per unit area (total nuclei/mm^2^).

To run the ViaFuse-V macro, open Fiji software and upload the desired image in “.lsm” format for stacked images or “.tif” format for unstacked images. If working with stacked images, unstack them (Image>Stacks>Stack to Images) and close all images except for the nuclei image. This is a crucial step to ensure the macro performs the analysis on the correct image. Run the macro on the image (Plugins>Macros>Run>Select Macro). The macro will prompt the user to select the folder where results will reside. Once the user has selected the desired folder, click “Open”. Another dialog box will appear. The first box will have the file path based on the previous selection. Click on the second box and type the desired name of the .csv file of the results. Click “OK” when finished. The program will then prompt the user for a multiplication value for the image being analyzed. A number will need to be typed and then click “OK” when done. The process will take a few minutes to finish. When the macro finishes the calculations, a “Calculations Complete!” message will be displayed. Click “OK” to exit the message. The results file can be found on the user’s computer where they chose to save it. This macro assumes that the units of the image are already in μm units. If this is not the case, the image needs to be converted accordingly. The numbers output from ViaFuse-V can be recorded into an excel that will generate the total number of nuclei (Supplementary Table 1).

### ViaFuse-F, macros to calculate fusion index

ViaFuse-F creates a mask of the MYH image and subtracts it from the DAPI image to generate an outside nuclei image with the nuclei in MYH-positive cells removed. ViaFuse-F uses this outside nuclei image to generate a .csv file that has (i) a list of the areas of particles greater than 251 μm^2^, (ii) the number of nuclei with areas between 50 μm^2^ and 250 μm^2^, and (iii) the mode of the areas of nuclei between 50 μm^2^ and 250 μm^2^. The number of nuclei that are in nuclear clumps outside of MYH-positive cells is determined by taking the sizes of nuclei greater than 251 μm^2^ that are outside of MYH-positive cells (generated by ViaFuse-F) and dividing them by the mode of the area of nuclei in the outside nuclei image sized between 50 μm^2^ and 250 μm^2^ (generated by ViaFuse-F). The number of nuclei in MYH-positive cells with less than three nuclei needs to be counted by the user. The total nuclei number from ViaFuse-V is used in the final fusion index equation. Fusion index is thus calculated using the following equation:

$$\mathrm{Fusion}\ \mathrm{in}\mathrm{dex}=\frac{\mathrm{total}\ \mathrm{nuclei}-\mathrm{nuclei}\ \mathrm{outside}\ \mathrm{MYH}\ \mathrm{positive}\ \mathrm{cells}-\mathrm{nuclei}\ \mathrm{in}\ \mathrm{MYH}\ \mathrm{positive}\ \mathrm{cells}\ \mathrm{with}\ 1\ \mathrm{or}\ 2\ \mathrm{nuclei}}{\mathrm{total}\ \mathrm{nuclei}}\times 100$$

To run the ViaFuse-F macro, open Fiji and upload the image in “.lsm” format. Ensure that the DAPI and MYH images are stacked together. Do not unstack the images. If other images are open besides the DAPI and MYH images this will not impact the macro. The macro assumes a specific stack order that is specified in the macro instructions available at the download link. Run the macro on the image (Plugins>Macros>Run>Select Macro). There will be a prompt for the user to select the folder to deposit results. Once the user has selected the desired folder, click “Open” and a dialog box will appear. The first box will have the file path based on the previous selection. Click the second box and type the desired name of the .csv file of the results. Click “OK” when finished. The program will then ask the user for a multiplication value for both the MYH image and DAPI image. Type in a number in each box, then click “OK” when done. The process will take a few minutes to finish. When the macro finishes the calculations, a “Calculations Complete!” message will be displayed. Click “OK” to exit the message. The .csv file can be found on the user’s computer in the folder where the user chose to deposit results. The merged .png image of the myotubes and nuclei counted can be found in the same location with the same name as the .csv file.  The numbers output from ViaFuse-F together with the manually counted nuclei in myotubes with 1 or 2 nuclei can be recorded into an excel that will generate the fusion index (Supplementary Table 1).

### Operation and guidelines for MyoCount usage

The images to be quantified using MyoCount were first divided into 3 × 3 montages using Fiji. The nine panels for each image were processed in a batch using the MyoCount program as previously described [11]. The following parameters were set for analysis: SmallestMyotubePixelCount = 200, SmallestNucleusPixelCount = 50, NucFillSize = 2, MaxCircleRad = 30–35, MaxNucSizeDivisor = 200, TubeThresh = 0.73.

### Testing

Researchers quantified total number of nuclei per unit area (mm^2^) and the fusion index of nine images of immunofluorescence-stained myotubes from three experiments using Fiji/ImageJ software (version 1.53c on MacOS, version 1.53 h on Windows), MyoCount program (version 1.3.1 with MATLAB Runtime Version 9.4 R2018a on Windows), and the manual image analysis methods described above. For the ViaFuse macros quantification, the default settings of the macros were used, and the multiplication values of each image were adjusted by the investigators. A Pearson correlation coefficient was calculated to compare values from the ViaFuse to those achieved manually or through the usage of the MyoCount program. All data analysis was performed using Microsoft Excel.

### Statistical analysis

Statistical analysis was performed in Microsoft Excel (Microsoft). Significance was determined using an unpaired Welch’s *t* test (two-tailed). The difference between two conditions was considered statistically significant when *p* < 0.05. All data are presented as the mean plus and minus the standard error from the mean (sem).

## Results

### Loss of CAPZB results in a trend of less cell viability and differentiation

To develop the ViaFuse macro package, the CAPZB protein was depleted using si-RNAs in C2C12 myoblasts, and skeletal muscle cell viability and differentiation were assayed. CAPZB caps the barbed end of actin filaments, preventing the addition or degradation of actin which is critical for ensuring constancy of actin filament length [12]. Global knockout of CAPZB in mice leads to embryonic lethality [13, 14], but the role of CAPZB in C2C12 cells has not been examined.

**Fig. 1:**
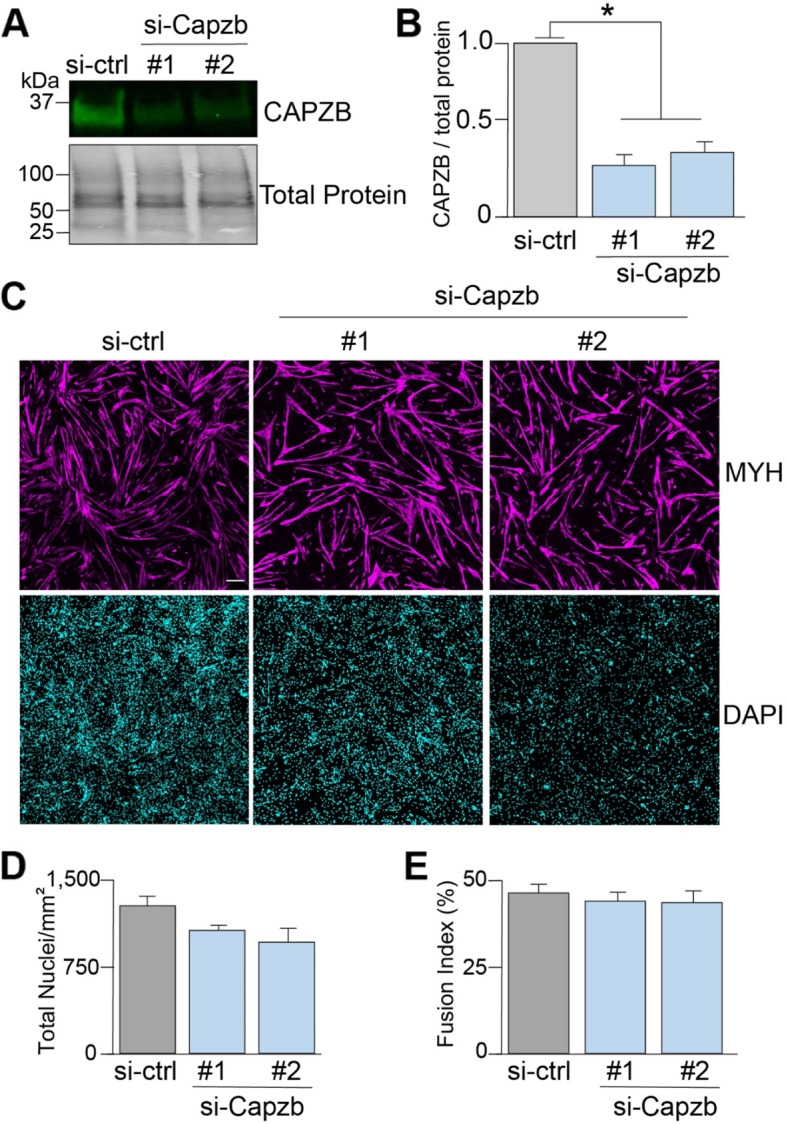
CAPZB depletion results in a trend of less cell viability and differentiation. CAPZB was depleted in myotubes using two different si-RNAs (#1 and #2), and a negative control si-RNA was used (si-ctrl). **A**, **B** Western blot assays were performed (**A**) and quantified by densitometry (**B**). **C** Immunofluorescence experiments were performed in myotubes after 4 days of differentiation. Cells were stained with DAPI (cyan) and an antibody recognizing the myosin heavy chain protein (MYH) (magenta). Scale bar = 200 μm. **D** The number of nuclei per unit area (mm^2^) was determined manually. **E** Muscle cell differentiation was determined by calculating the fusion index manually. Results are shown as mean + s.e.m., *p < 0.05, Welch’s *t* test, *n* = 3 independent experiments

The CAPZB protein was significantly knocked down in cells treated with two different si-RNAs (#1 and #2) compared to the negative control (Fig. 1A, B). Cells were stained with DAPI (which stains nuclei) and with an antibody recognizing the myosin heavy chain protein (MYH), which is a marker of muscle cell differentiation (Fig. 1C). Total nuclei can be counted to infer levels of cell viability. The fusion index is indicative of the level of differentiation from myoblasts into myotubes [3, 5] and is defined as the proportion of nuclei in MYH-positive cells with three or more nuclei with respect to the total number of nuclei in a field of view. Manual calculations revealed a trend of less total number of nuclei per unit area (total nuclei/mm^2^) (Fig. 1D) (si-ctrl: 1280 ± 87, #1: 1068 ± 36, #2: 960 ± 125, si-ctrl vs #1: *p* = 0.1, si-ctrl vs #2: *p* = 0.1) and only slightly less fusion index for both si-RNAs in comparison with the controls (Fig. 1E) (si-ctrl: 47 ± 3, #1: 44 ± 2, #2: 44 ± 3, si-ctrl vs #1: *p* = 0.6, si-ctrl vs #2: *p* = 0.5). This indicates that CAPZB could be important in skeletal muscle cell viability but less relevant for myoblast differentiation into myotubes.

### The ViaFuse-V macro estimates muscle cell viability

**Fig. 2:**
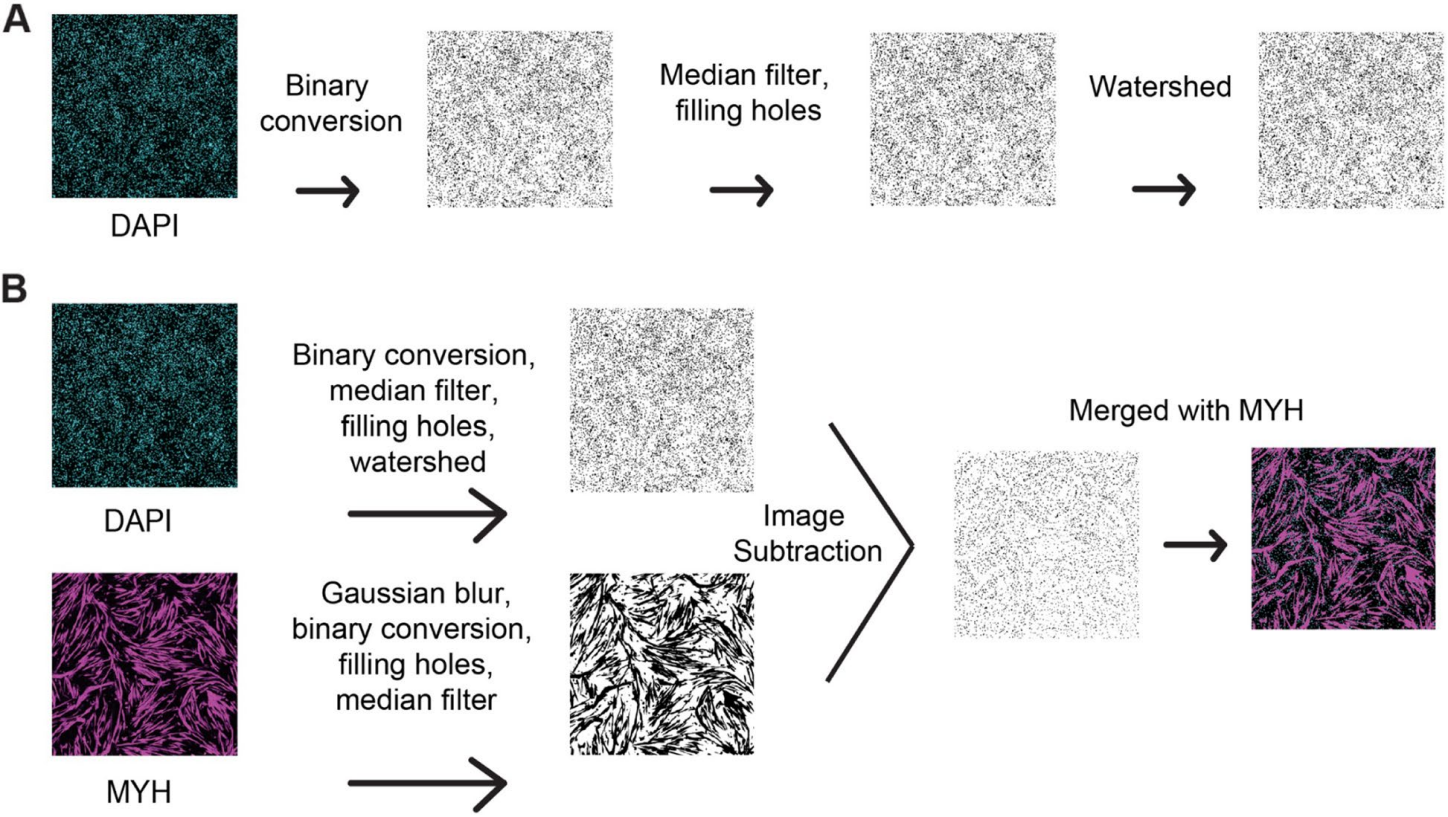
ViaFuse macro implementation steps. **A** The first image in cyan was the original DAPI image before any analyses. The second image was generated after thresholding to make the image binary. The third image was generated after the application of a median filter and the filling of holes. The final image was generated by applying a watershed step. **B** The first image in cyan was the original DAPI image before any analyses. The same steps described in **A** were taken to achieve the next DAPI image. The MYH image (original) in magenta underwent the same analyses as the DAPI image. The final images were subtracted from one another resulting in a composite of DAPI and MYH after subtraction

ViaFuse-V fully automated the steps that were used above for manual quantification in the Fiji image analysis software. To determine the total number of nuclei in a field of view, this macro first multiplied the image by the user input to make it easier to identify the nuclei and their approximate outlines. Then, ViaFuse-V applied a threshold to the image using the default method in Fiji to make it a binary image (Fig. 2A and Table 1). Next, ViaFuse-V applied a median filter to reduce image noise followed by closing the small holes and the nuclei were further watershed segmented to identify their center points (Fig. 2A and Table 1). Finally, to retrieve relevant nuclei data for analysis, ViaFuse-V analyzed the image for particles in two steps: (i) it counted the particles ranging between 50 μm^2^ and 250 μm^2^ in area and (ii) counted the particles larger than 251 μm^2^ (considered “clumps of nuclei”) (Table 1). To calculate the total number of nuclei in a field of view, the area of each nucleus larger than 251 μm^2^ (generated by ViaFuse-V) was divided by the mode of all nuclei with areas between 50 μm^2^ and 250 μm^2^ (generated by ViaFuse-V) to determine the number of nuclei in the large clumps. We then added this number to the total number of nuclei with areas between 50 μm^2^ and 250 μm^2^ (generated by ViaFuse-V) to procure the total number of nuclei in the field. Finally, we divided the total number of nuclei found by the area of the image (measured in mm^2^ and generated by ViaFuse-V) to obtain the total number of nuclei per unit area.

**Table 1:** Analysis of the total number of nuclei analysis using ViaFuse-V: parameter and default settings. The purpose of each parameter and its function is explained in the description column and the default setting of that parameter is shown

Parameter	Default setting	Description
Multiplication	1	Multiplies the image by the specified value. The default value of 1 indicates that there is no multiplication. Value must be greater than 1 to increase the brightness of the image. Useful when image is too dark. User is prompted to change this setting if desired
Median filter	1 pixel	Reduces noise in the image by replacing each pixel with the median of the neighboring pixel values. User is not prompted to change median radius value but may do so in the macro script
Analyze particles step 1	50–250 μm^2^	Analyzes DAPI image for nuclei of these areas to obtain number of nuclei. User is not prompted to change this setting but may do so in the macro script
Analyze particles step 2	251 μm^2^–infinity	Analyzes DAPI image for nuclei of these areas to obtain the areas of nuclei found in clumps. User is not prompted to change this setting but may do so in the macro script

### ViaFuse-F calculates muscle cell fusion index

Next, ViaFuse-F was developed to calculate the cell fusion index. To automate the process of counting nuclei outside MYH-positive cells for fusion index calculations of both the DAPI and MYH images were utilized. The DAPI image was analyzed using the same process as described in the previous section to obtain the total number of nuclei in a picture. To count the nuclei outside of MYH-positive cells, ViaFuse-F first identified myotubes and their outlines by smoothing the image using the Gaussian Blur filter followed by applying a threshold to the image using the default method in Fiji to make it a binary image and then a median filter to reduce the noise and then filled the small holes (Fig. 2B *left* and Table 2). The MYH image was next subtracted from the nuclei image, which produced the image of the nuclei outside of MYH-positive cells (Fig. 2B *right* and Table 2). To retrieve those nuclei outside the MYH-positive cells for analysis, the resulting image from the subtraction was analyzed for particles with areas: (i) between 50 μm^2 ^and 250 μm^2^ and (ii) greater than 251 μm^2^. The area of particles (clumps) greater than 251 μm^2^ was divided by the mode of the areas of all nuclei with areas between 50 μm^2^ and 250 μm^2^ to determine the number of nuclei in the large clumps. Therefore, to retrieve the number of nuclei outside the MYH-positive cells the user adds this number to the total number of nuclei with sizes between 50 μm^2^ and 250 μm^2^ to procure the number of nuclei outside MYH-positive cells.

**Table 2:** Calculation of the fusion index using ViaFuse-F: parameter and default settings. The purpose of each parameter and its function is explained in the description column and the default setting of that parameter is shown

Parameter	Default setting	Description
Multiplication	1	Multiplies each of the images by the specified value. The default value of 1 indicates that there is no multiplication. Value must be greater than 1 in order to increase the brightness of the image. Useful when image is too dark. User is prompted to change this setting if desired
Median filter	1 pixel	Reduces noise in each of the images by replacing each pixel with the median of the neighboring pixel values. User is not prompted to change median radius value but may do so in the macro script
Gaussian blur (sigma)	2 pixels	Smooths MYH image by giving a higher weight to edge pixels than pixels inside the image, making the myotube outlines clearer. User is not prompted to change the sigma value but may do so in the macro script
Analyze particles step 1	50–250 μm^2^	Analyzes DAPI image for nuclei of these areas to obtain number of nuclei. User is not prompted to change this setting but may do so in the macro script
Analyze particles step 2	251 μm^2^–infinity	Analyzes DAPI image for nuclei of these areas to obtain the areas of nuclei found in clumps. User is not prompted to change this setting but may do so in the macro script

To calculate the fusion index, the nuclei outside of MYH-positive cells (counted with ViaFuse-F) and those inside MYH-positive cells with one or two nuclei (manually counted) were added together, subtracted from the total number of nuclei (obtained by ViaFuse-V), and divided by the total number of nuclei in the field (obtained by ViaFuse-V) and multiplied by 100. We have adopted a strict criterion of differentiation and do not consider cells expressing MYH with only one or two nuclei as myotubes. In this manner, the fusion index indicates the proportion of nuclei in myotubes (considered as those MYH-positive cells with three or more nuclei) versus all the nuclei in the image.

### Quantifications with ViaFuse have high correlation to manually quantified values

**Fig. 3:**
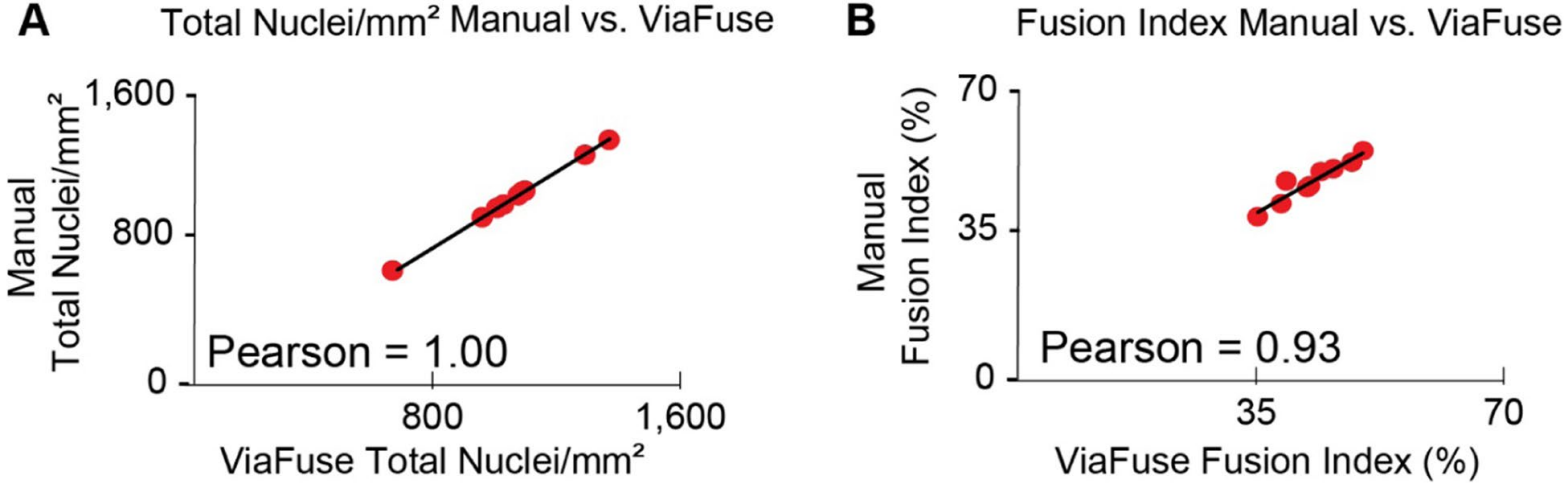
Quantifications with ViaFuse have high correlation to manually quantified values. **A** Graph of total nuclei per unit area (mm^2^) as quantified by ViaFuse-V compared to the manual quantification. **B** Graph of the fusion index as quantified by ViaFuse-F compared to the manual quantification. The Pearson correlation coefficient was calculated to compare the different methods

The ViaFuse-V macro was next used to analyze the same images as those analyzed manually and the outcomes were compared. The ViaFuse-V macro produced the same total nuclei per unit area values as those found manually (Pearson = 1) (Fig. 3A), thus it is a dependable way to measure the number of nuclei. For fusion index, ViaFuse-F produced similar values as those found manually, with a high correlation coefficient (Pearson = 0.93) (Fig. 3B), indicating that it is also an accurate tool to measure fusion index.

### ViaFuse quantifications highly correlate with those from MyoCount

**Fig. 4:**
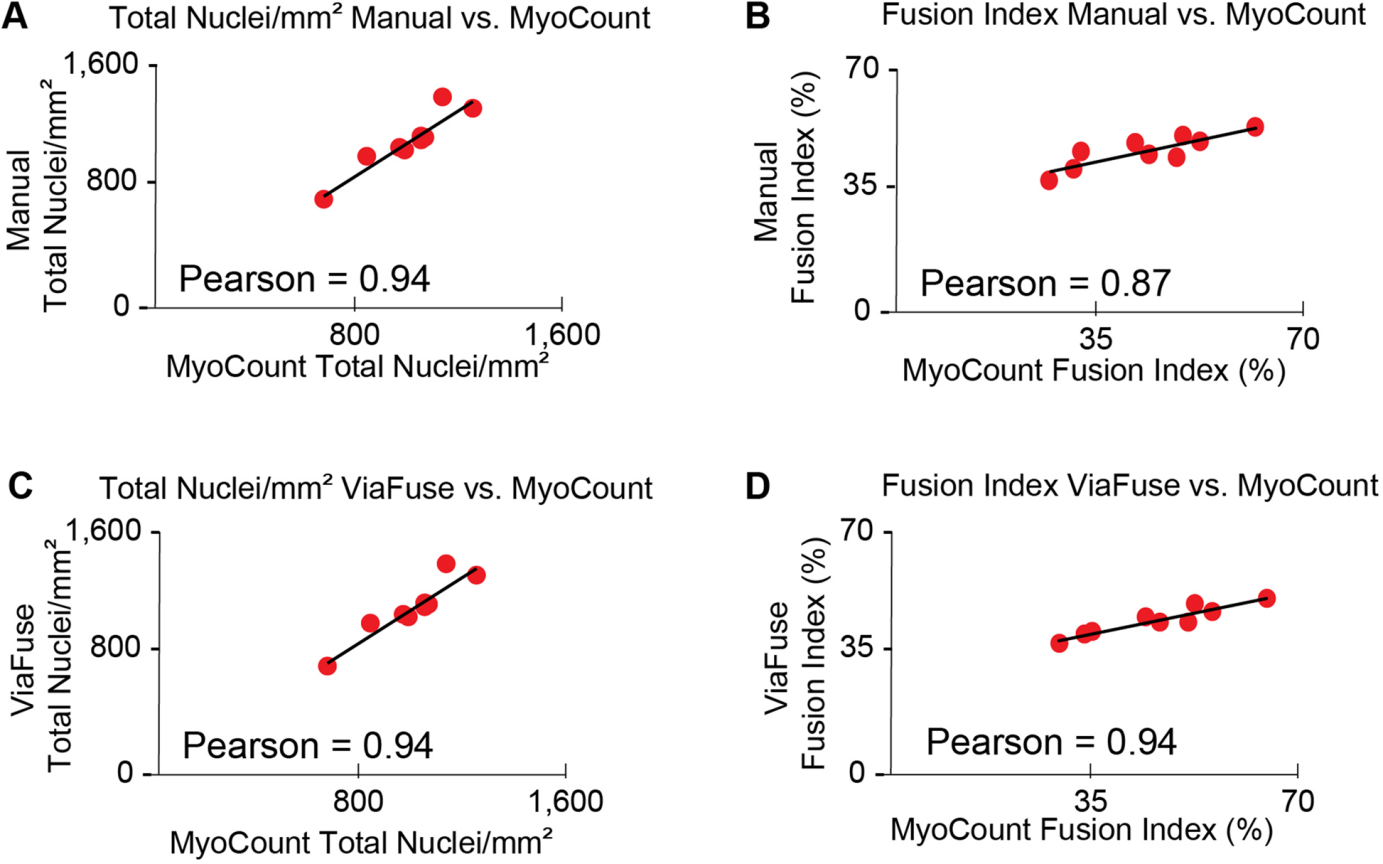
ViaFuse quantifications highly correlate with those from MyoCount. **A** Graph of total number of nuclei per unit area (mm^2^) as quantified by MyoCount compared to the manual quantification. **B** Graph of the fusion index as quantified by MyoCount compared to the manual quantification. **C** Graph of total number of nuclei per unit area (mm^2^) as quantified by the ViaFuse-V compared to MyoCount quantification. **D** Graph of the fusion index as quantified by ViaFuse-F compared to MyoCount quantification. The Pearson correlation coefficient was calculated to compare the different methods

To assess the accuracy of the ViaFuse macros, the quantifications obtained with ViaFuse were compared to the results from the existing software tool MyoCount [11]. We first confirmed that MyoCount resulted in similar total nuclei per unit area values as those found manually (Pearson = 0.94) (Fig. 4A) and similar fusion index values as well (Pearson = 0.87) (Fig. 4B). Next, we compared ViaFuse results with those obtained using the MyoCount program. We found that ViaFuse produced similar values for the total number of nuclei per unit area and the fusion index as those obtained using MyoCount, with a Pearson correlation coefficient of 0.94 (Fig. 4C) and 0.94 respectively (Fig. 4D).

### ViaFuse overcomes limitations of MyoCount

**Fig. 5:**
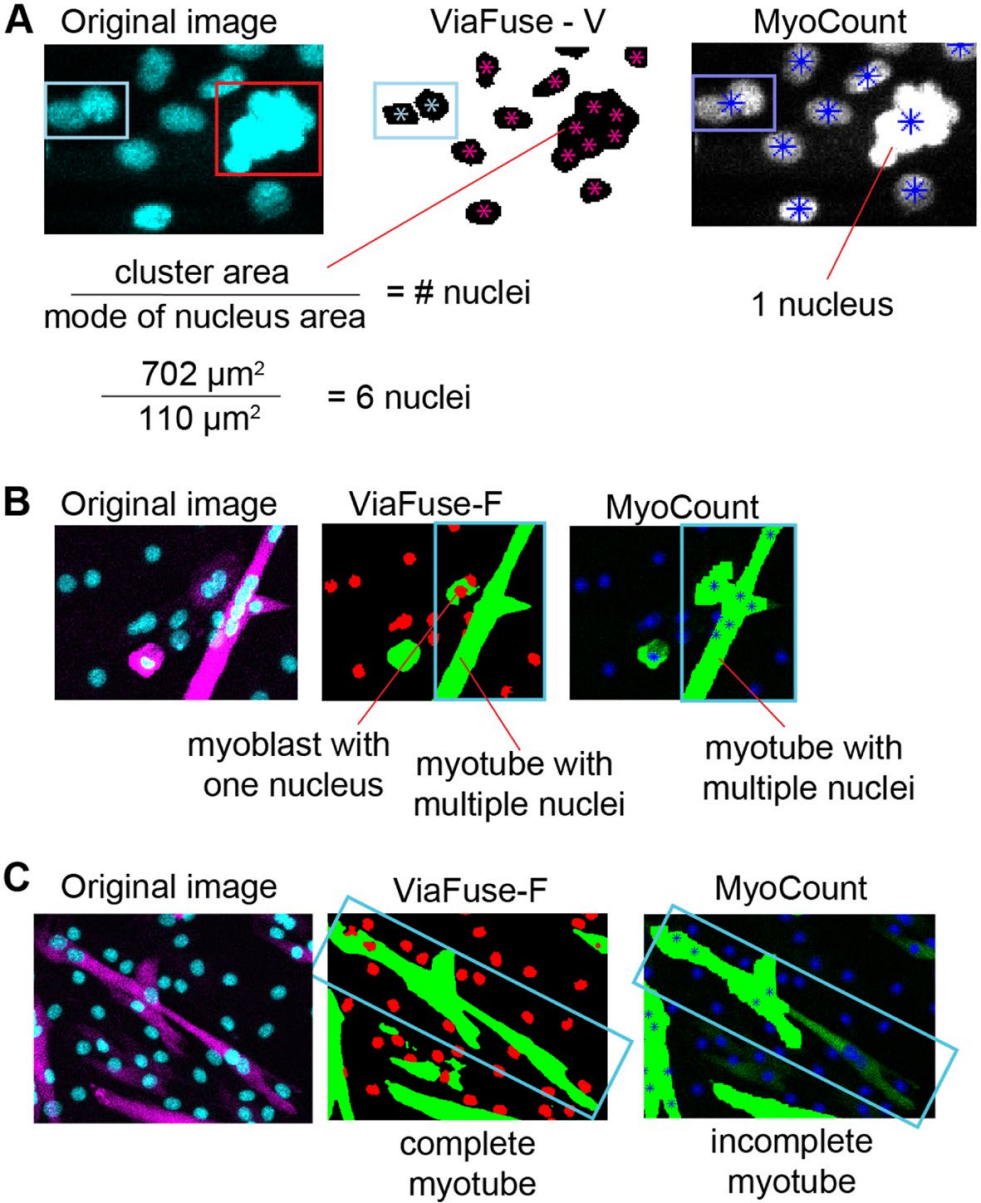
ViaFuse overcomes limitations of MyoCount. **A** Images demonstrating how ViaFuse-V can more accurately determine nuclear clumps compared to MyoCount by (a) applying a watershed step (light blue rectangle) and (b) estimating the number of nuclei in particles greater than 251 μm^2^ by dividing their sizes by the mode of the nuclei sized 50–250 μm^2^. **B** ViaFuse-F distinguished myoblasts that are close to myotubes. **C** Images demonstrating that ViaFuse-F defined myotubes borders accurately in situations where MyoCount considered them incompletely

We notice several limitations when using MyoCount. First, in numerous cases MyoCount does not properly identify individual nuclei when they are clumped together, thus, considering multiple clumped nuclei as one nucleus (Fig. 5A *right*). In contrast, ViaFuse-V accurately identifies a more exact number of nuclei in a clump. This is due to two steps we utilized in our macros. First, ViaFuse-V more accurately employs watershed to divide particles that are larger that might contain more than one nucleus (based on size and shape) (Fig. 5A *left*, light blue rectangle), while MyoCount would consider that particle as one nucleus (Fig. 5A *right*), ViaFuse-V divided it and computed it as two nuclei (Fig. 5A *middle*, light blue rectangle). Second, ViaFuse-V included one step of computing the area of clumps of nuclei (particles with a size > 251 μm^2^) and the user divided this number by the mode of the areas of all nuclei sized between 50 μm^2^ and 250 μm^2^. In this manner, a cluster of nuclei (Fig. 5A *left*, red rectangle) that was considered as only one nucleus by MyoCount (Fig. 5A *right*) was estimated to contain approximately six nuclei by ViaFuse-V (Fig. 5A *middle*).

A second important problem of MyoCount is that in some situations it considered individual myoblasts as part of a neighbor myotube (Fig. 5B *right*). When this happened, those myoblast nuclei were counted as part of differentiated myotubes leading to an overestimation of the fusion index. ViaFuse-F, in turn, overcame this problem by distinguishing close myoblasts from myotubes (Fig. 5B *middle*).

Third, and related to the previous point, we observed that in some cases MyoCount was not able to distinguish the borders of multiple myotubes that are close to each other or multiple myoblasts that are adjacent (but clearly not fused). This was a concern especially when measuring the fusion index.

Finally, in multiple images, we noticed that MyoCount identified incomplete myotubes (Fig. 5C *left* versus *right*) whereas ViaFuse accurately determined the full outline of myotubes (Fig. 5C *left* versus *middle*).

## Discussion

In this paper, we compared three methods of quantifying skeletal muscle cell viability and fusion: manual counting, the MyoCount program [11], and our newly developed ViaFuse macros. Overall, ViaFuse performs similarly to the other quantification methods in terms of the results, but its accessibility, ease of use, and accuracy in resolving nuclear clumps, distinguishing myoblasts from myotubes in close proximity, and identifying myotube borders make it superior.

Manual quantification of skeletal muscle cells is very accurate, but is extremely time-consuming and researchers should be blinded to avoid counting bias. The MyoCount software attempted to address the time-limiting factor of skeletal muscle cell quantification by automating the process of determining the total number of nuclei in microscopy images and the fusion index value. However, this software is still severely limited by its inability to (a) resolve nuclear clumps, (b) distinguish myoblasts from myotubes in close proximity, (c) outline myotubes in multiple situations, as well as by (e) the heavy equipment requirement (lot of computer RAM), and (f) the need of a deep mathematical knowledge of MATLAB by the user.

The differentiation of myoblasts into myotubes is marked by the fusion of multiple myoblasts together which often results in nuclear clumping. Various treatments or culture conditions of myoblasts can also promote nuclear clumping by affecting cell-cell fusion. Since MyoCount is unable to distinguish nuclei that are in a nuclear clump this leads to an underestimation of the nuclei in clumps and an inflation of the fusion index.

MyoCount also has difficulty estimating the borders of myotubes or myoblasts that are close together which is a phenotype that can naturally occur during muscle cell differentiation [11]. This results in multiple close myoblasts being counted as one myotube which further inflates the fusion index. MyoCount can count MYH-positive cells with less than three nuclei in them to generate fusion index automatically; however, this number is not fully accurate because of the aforementioned issues with resolving nuclear clumps, counting myoblasts as myotubes, and defining myotube borders. Further, MyoCount applies the same counting parameters to every image; so, if a treatment or condition significantly changes myotube morphology, or leads to more nuclei clumping, MyoCount is unable to resolve these changes.

ViaFuse addresses both the time limitations of manual counting and the inability of MyoCount to resolve nuclear clumps and proper myotube and myoblast borders. ViaFuse can precisely break down clusters of nuclei into exact numbers based on their area. When images are quantified by hand, the discretion lies with the researcher, who decides how many nuclei are in a clump, which can lead to a more variable count as has been identified by other groups [11]. Indeed, the differences in fusion index values between ViaFuse and manual quantification can be attributed to the fact that ViaFuse more accurately counts nuclei in clumps. In the quantifications performed using MyoCount, we observed a trend of higher fusion index compared to both manual and ViaFuse quantifications which is partly due to inability to identify nuclei in a clump. When comparing total nuclei images from ViaFuse and MyoCount, ViaFuse accurately determines the exact number of nuclei in the clump.

ViaFuse calculates the fusion index by computing the nuclei outside of myotubes with three or more nuclei (and then subtracts this number from the total number of nuclei) whereas MyoCount calculates the fusion index by counting nuclei inside of myotubes with three or more nuclei [11]. Counting nuclei outside of myotubes is more precise because it is easier to quantify nuclear clumps, and there is higher contrast between the black background and nuclei signal than between the background and the merged signal from MYH and DAPI images. One important parameter that ViaFuse-F asks the user to adjust is the brightness of the MYH and the DAPI images. It is important for the MYH image to be brighter than the DAPI image so that the nuclei are subtracted properly from the MYH image. It is crucial that the researcher standardizes the brightness levels of the DAPI and MYH images within experiments and before analysis to avoid drastic differences. This step cannot be automated because it is highly dependent on the microscopy images. Assuming that all the MYH images were taken with the same acquisition parameters (as should be the case) many times the DAPI images can exhibit brightness differences due to minimal variations in the focal plane across images and/or conditions. The best way to standardize the brightness levels for DAPI and MYH images is consciously by the researcher. It is also something to consider during the image acquisition.

To overcome the limitation of skeletal muscle cell biology which involves nuclear clumping and overlapping myotubes and/or unfused myotubes as a product of the differentiation course, we leveraged automation as well as manual counting for accurate fusion index estimation. Software is unable to parse out MYH-positive cells with less than three nuclei so with ViaFuse we count the nuclei in these cells manually to generate the most accurate value of the fusion index. Indeed, using MyoCount results in fusion index inflation due to underestimation of total nuclei and considering several myoblasts or myotubes as one myotube. Thus, ViaFuse is superior because it combines the efficiency of automation with the high accuracy of manual counting.

Beyond biological accuracy, ViaFuse-V and ViaFuse-F are easy to run since they are Fiji plugins and users can perform analyses quickly without requiring a computer with lots of RAM. MyoCount can often be difficult to troubleshoot unless the user is very familiar with MATLAB code. In summary, ViaFuse is a robust replacement of MyoCount and addresses its limitations.

## Conclusions

The ViaFuse macro package provides users with a quick and easy way to analyze nuclei in a field of view as well as muscle cell fusion by interfacing with Fiji and requiring minimal user input. Further, ViaFuse can distinguish neighboring myotubes and myoblasts as well as clumped nuclei which is a limitation of current software. We anticipate that the ViaFuse macros will save researchers a great amount of time analyzing skeletal muscle cell viability and differentiation as well as produce robust results that accurately represent the biology.

### Availability and requirements

Project name: ViaFuse.

Project home page: https://github.com/tasneemsmacros/ViaFuse

Operating system(s): Windows XP or later, Mac OS X 10.8 “Mountain Lion” or later, or a Linux on amd64 and × 86 architectures.

Programming language: ImageJ Macro Language (IJM).

Other requirements: Java 8 or higher.

License: None.

Any restrictions to use by non-academics: None.

## Supplementary Information

**Additional file 1: Supplementary Table 1**.

## Data Availability

The datasets and materials used and/or analyzed during the current study are available from the corresponding author on reasonable request.

## Abbreviations

BIF: Buffer for immunofluorescence
CAPZB: Capping actin protein of muscle z-line beta
DAPI: 4′,6-diamidino-2-phenylindole
DMEM: Dulbecco’s modified Eagle’s medium
Fiji: Fiji is just ImageJ
LMNA: Lamin A/C
MATLAB: Matrix laboratory
MYH: Myosin heavy chain
PBS: Phosphate-buffered saline
RAM: Random access memory
rpm: Revolutions per minute
SDS: Sodium dodecyl sulfate
sem: Standard error of the mean
si-RNA: Small interfering RNA
TBST: Tris-buffered saline with tween
ViaFuse: Viability and fusion
ViaFuse-F: ViaFuse fusion
ViaFuse-V: ViaFuse viability
